# Role of Dermatix in the Management of Eyelid Hypertrophic Scars After Facial Trauma

**DOI:** 10.7759/cureus.61450

**Published:** 2024-05-31

**Authors:** Cristiana Germano, Carlo Calvanese, Giovanni Dell' Aversana Orabona, Vincenzo Abbate, Paola Bonavolontà

**Affiliations:** 1 Maxillofacial Surgery Operative Unit, Department of Neurosciences, Reproductive, and Odontostomatological Sciences, University of Naples Federico II, Naples, ITA

**Keywords:** cosmetic outcome, eyelid elevator, hypertrophic scar, head and neck trauma, dermatix silicone gel

## Abstract

Facial trauma can cause skin wounds with uneven and discoloured edges that require healing by secondary intention. These wounds often produce excess collagen fibres, leading to fibrosis and hypertrophic scars that can cause discomfort and negatively impact the patient's quality of life.

A man suffered facial trauma due to a motor vehicle accident, resulting in a fracture of the left zygomatic-maxillary complex. He underwent surgery to fix the fracture and reconstruct his eyelid but developed a hypertrophic scar during recovery that caused eye dryness and discomfort. To treat the scar, Dermatix silicone gel (SG) (Viatris, Canonsburg, PA) was applied twice a day. After two months of treatment, the scar had improved significantly, and the patient's eyelid function had also improved.

This case describes the use of Dermatix SG to treat a patient with a traumatic hypertrophic scar of the eyelid associated with eyelid malposition. Silicone gel is a non-invasive treatment for scars and has been shown to be effective in reducing scar elevation and erythema. However, there is a gap in the literature regarding the routine use of SG to preserve functionality and aesthetics in traumatic hypertrophic scars of complex anatomical structures. Further studies are needed to understand the principles of using SG for these types of scars to improve functional and aesthetic outcomes.

Applying Dermatix SG twice a day for 60 days corrected a patient's functional and aesthetic issues. More studies should be conducted to investigate the product's effectiveness further.

## Introduction

Facial trauma often results in lacerations and contusions, leading to irregular, finely fringed, and ecchymotic skin margins, which impede normal healing and often result in scar formation. The immature stratum corneum of the wound exacerbates this process, leading to excessive transepidermal water loss and increased collagen production. Mechanical forces on the wound further induce collagen allocation, resulting in fibrosis and hypertrophic scars. These scars can significantly impact a patient's quality of life, causing discomfort and even leading to depression and anxiety [1]. An aesthetic insult is often associated with functional damage in several facial areas, such as the eyelid, due to thin skin and the complex anatomical structure. Lacerated and contused eyelid wounds may stretch or disrupt the eyelid elevator muscle. The healing process will lead to fibrosis and retraction of the tissues overlying the muscle, resulting in reduced eyelid motility [2].

Among the several available treatment options, topical silicone gel (SG) treatments are ranked first in the international recommendations for scar management, according to the International Advisory Panel on Scar Management [3, 4]. Silicone gel provides hydration and reduces hypertrophic scarring. Dermatix (Viatris, Canonsburg, PA) is a Food and Drug Administration (FDA)-registered equivalent to SG. The application of Dermatix gel flattens, softens, and smoothes the skin, preventing and managing hypertrophic scars and keloids [5]. Although Dermatix SG is an FDA-tested product approved for the prevention of hypertrophic scars, keloids, and surgical wounds, there is currently no study in the literature addressing the use of Dermatix to improve the functional status of muscles damaged by trauma. Our study aimed to describe the aesthetic result and functional improvement of an eyelid hypertrophic scar, determining contracture in the eyelid movement by applying Dermatix to a facial trauma patient [1]. A review of the existing literature on SG adoption in the scar’s treatment is performed. Hopefully, this case study will determine the increasing use of Dermatix SG in traumatic scars with muscle damage for functional improvement besides cosmetic.

## Case presentation

A 34-year-old man presented at the Department of Maxillofacial Surgery of our hospital, complaining of facial trauma due to a motor vehicle accident. In history, the patient did not report any healing, rheumatologic, or neurological disorders, which would have impaired the routine healing of the lacerated, contused wounds.

**Figure 1:**
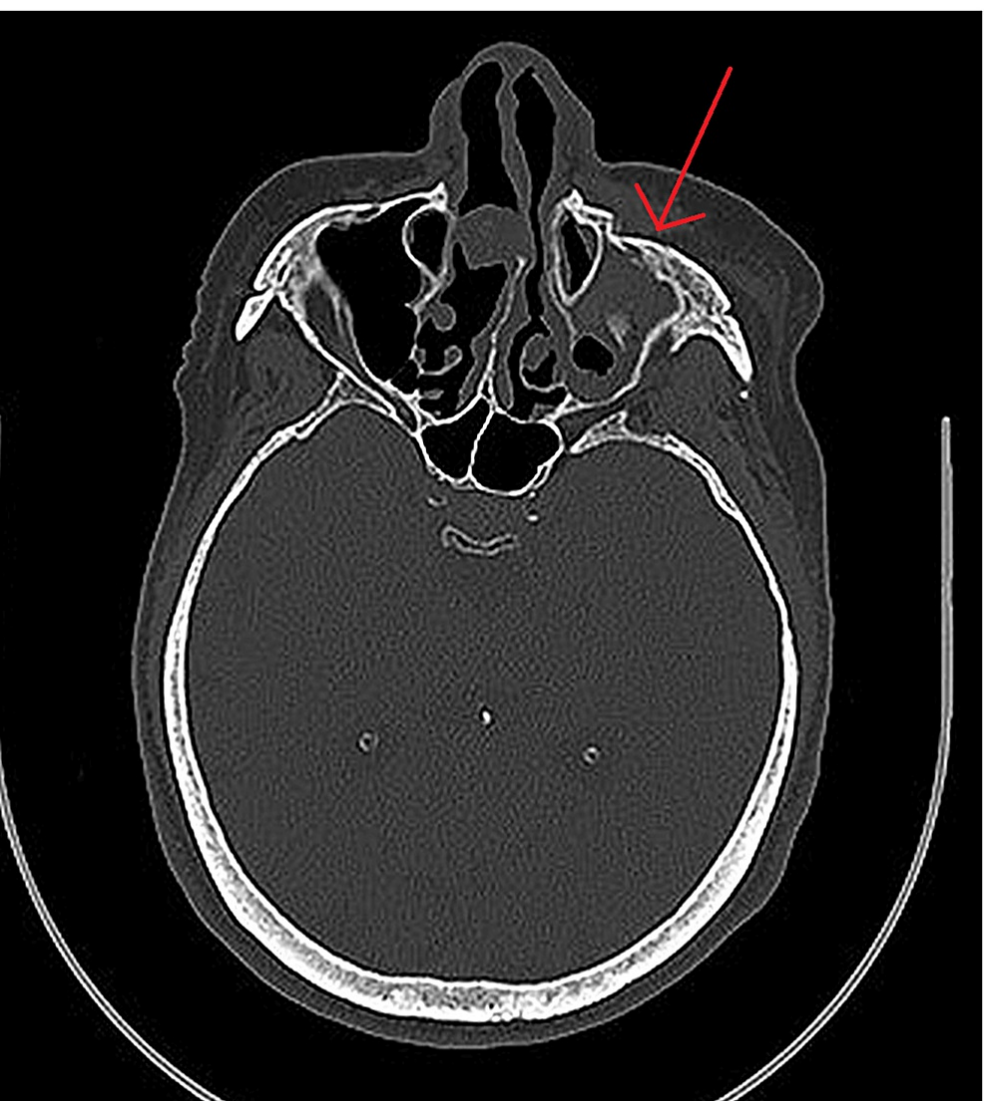
Preoperative CT scan Red arrow: left zygomatic-maxillary complex fracture

The computed tomography scan showed a fracture of the left zygomatic-maxillary complex (Figure 1).

**Figure 2:**
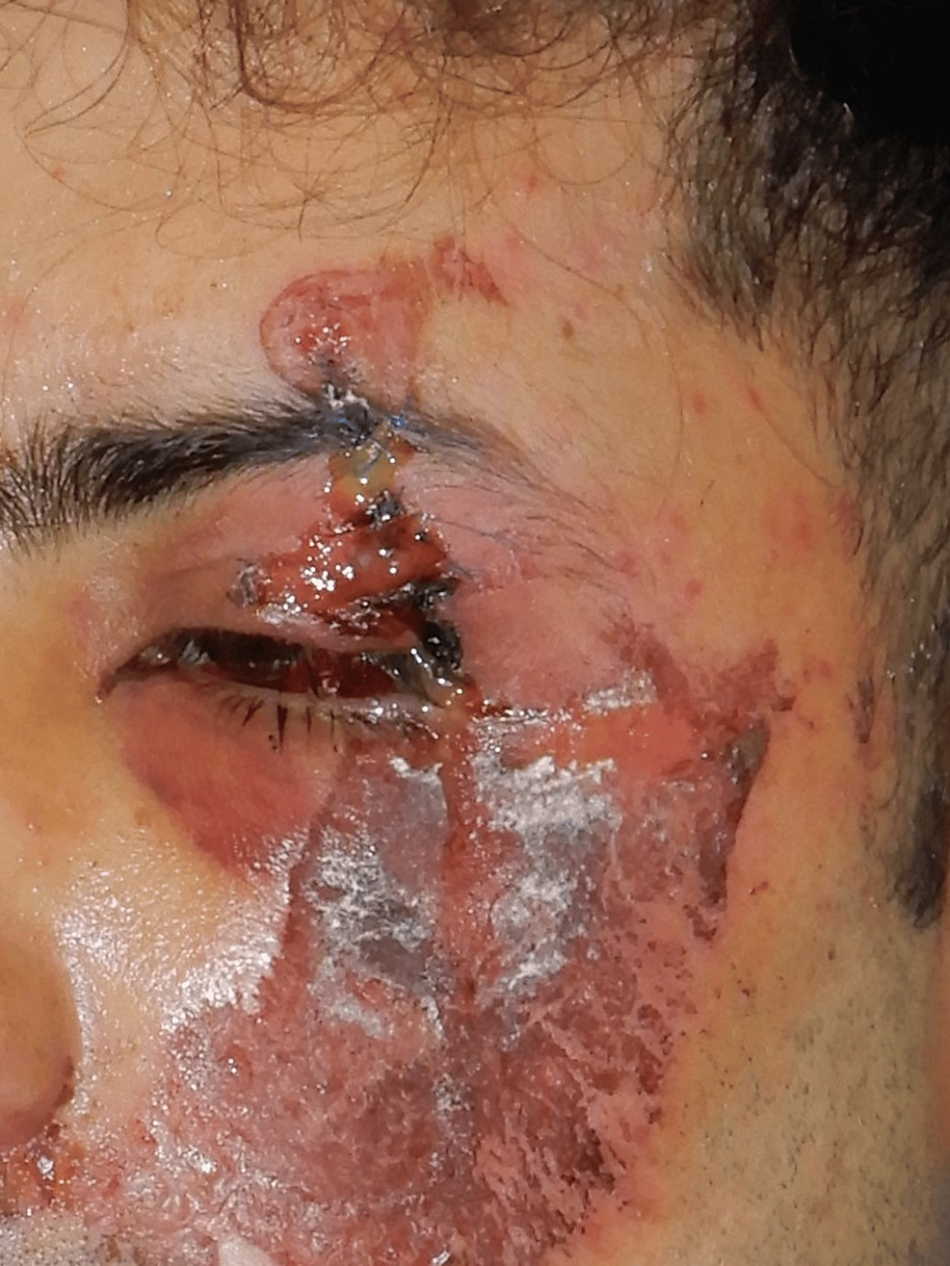
Preoperative picture of the left upper eyelid and eyebrow laceration wound

The patient presented a left upper eyelid and eyebrow laceration wound with significant damage to the skin and the eyelid elevator muscle (Figure 2).

Fracture open reduction and internal titanium fixation were performed, and surgical reconstruction of the eyelid was made by suturing the subcutis with Vicryl 5.0 (Ethicon Inc., Raritan, NJ) and an intradermal suture with nylon 6.0.

During the first postoperative month, the patient presented a hypertrophic scar associated with retraction of the upper eyelid, determining lagophthalmos. The patient complained of eye dryness, burning, foreign body sensations, and psychological discomfort. Due to scar retraction, closing the eyelid was impossible, exposing the cornea and excessively evaporating the tear film. This might lead to keratitis, corneal abrasion, infection, and endophthalmitis.

On the 30th day after the trauma, we decided to apply Dermatix SG to massage the eyelid hypertrophic scar, avoiding contact with the eye. The treatment protocol ranged from 10 to 15 minutes, twice daily, for two months. During this treatment period, the patient was instructed to use artificial tears to avoid a burning sensation and dehydration of the conjunctiva, which, over time, could have potentially produced infections in the tissues of the orbital cavity.

The Vancouver Scar Scale (VSS) was adopted to detect the wound changes, which includes the following assessment parameters: vascularization, pigmentation, flexibility, and height, with an increasing score from 0 to five as the degree of hypertrophy increases.

The functionality of the eyelid elevator muscle (the difference between opening and closing the eyelid) was measured in millimetres. An average difference was considered more than 15 mm, good between 12 and 14, weak between 11 and five, and poor between five and one.

At the first evaluation, one month after surgery (T0), the patient had a total VSS score of eight and an eyelid elevator muscle function of 12 mm. After 60 days (T1) of the treatment mentioned above, the total VSS score had decreased to two (Table 1).

**Table 1:** The VSS score of the patient at T0 and T1 T0: one month after surgery; T1: 60 days after surgery; VSS: Vancouver Scar Scale

Timeline	Vascularisation	Pigmentation	Flexibility	Height	Total
T0	1	2	4	1	8
T1	0	1	1	0	2

**Figure 3:**
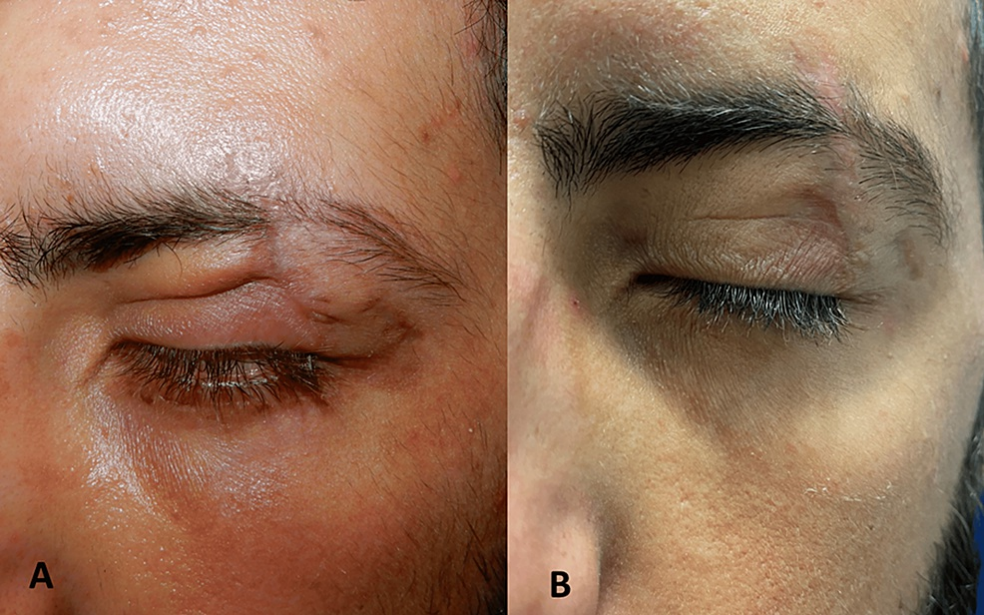
A. scar before treatment with Dermatix SG; B. scar after treatment with Dermatix SG SG: silicone gel

The function test improved by 2 mm, with an overall functionality of 14 mm. In addition, the ophthalmic evaluation confirmed the complete resolution of the complained keratopathy (Figure 3).

## Discussion

The present case describes an alternative use of SG to preserve the eyelid function in cases of hypertrophic scars, besides aesthetics, in a patient with facial trauma from a car accident. To our knowledge, there is no evidence of comparable SG utilisation in the literature.

Although the epidermis regenerates after trauma, the immature stratum corneum of the wound allows excessive transepidermal water loss, which induces fibroblasts to release more collagen fibres [6]. This induces scar retraction, which often causes a significant reduction in the patient's quality of life due to both aesthetic and functional outcomes [7]. The development of hypertrophic scars is even more frequent when they are associated with irregular margins and tissue loss due to trauma, especially when complex anatomical structures, such as those of the eyelid region, are affected [8]. In our case, not only was the thin and delicate skin of the eyelid affected, but there was also a rupture of the underlying tissue and, thus, an impairment of eyelid function. The pathological healing of the wound was followed by scar retraction and reduced eyelid mobility, which was very disabling for the patient. This condition is followed by lagophthalmos, which can potentially lead to infections of the conjunctiva and eyeball. Thus, ours is a case of aesthetic, functional, and medical impairment.

The idea of using an SG to treat hypertrophic scar tissue derives from its usability, reproducibility, and non-invasiveness. Several treatments exist to optimise scar formation during the healing process, including dressings to minimise wound tension, topical treatments, resurfacing, lasers, and intralesional injections of corticosteroids or botulinum toxin A.

A prospective randomised case-control study of 24 patients found no statistically significant difference between silicone gel and placebo for scar treatment after direct brow lift surgery [6]. However, it mentions surgical sutures for elective procedures. No mention is made of trauma patients. These differ in wound quality besides the orientation and cruentation of the underlying soft tissue.

On the other hand, another fascinating study by Yun et al. described 80 patients treated with a topical silicone gel mixture containing vitamin C, which achieved a significant decrease in scar elevation (p = 0.026) and erythema (p = 0.025) compared to the placebo control group [9].

An article published in 2022 also compared the scar treatment outcomes of the AnsCare LeniScar silicone stick and the conventional silicone gel Dermatix Ultra. There were no statistically significant differences between the two products. However, the former demonstrated the advantages of time-saving and product waste-sparing [10].

Recently, several innovative techniques for post-surgical scar management have been reported. Lim et al. described the embrace device, which creates a tension shield around the wound, promoting healing with minimal fibrosis and scarring. This device demonstrated a highly significant improvement in the appearance of scars after six months [11].

In a pilot study, Murison et al. demonstrated the efficacy of Dermatix in reducing the elevation and pigmentation of scars and 96.4% patient compliance with a twice-daily application [12].

Nevertheless, there seems to be insufficient evidence in the literature for the routine use of Dermatix SG to preserve functionality and aesthetics.

Although there are innovations in the medical field, topical treatments with silicone gel rank first in international recommendations for scar management, according to the International Advisory Panel on Scar Management [4]. There are several studies on the SG treatment of pathological scars. Still, there is a gap in the medical literature regarding SG management of traumatic hypertrophic scars of the eyelid associated with hypomotility due to elevator muscle involvement. This gap gave rise to the idea of describing the results obtained in terms of aesthetic and functional improvement of the retracting hypertrophic scar of our patient's eyelid with local application of silicone gel.

The majority of the evidence emphasises the surgical patient and anatomically non-complex tissues. To our knowledge, no study has described a different strategic use of SG. In our opinion, the product deserves more attention in this regard, as it is potentially helpful in avoiding primary corrective surgery, intended both to preserve the aesthetics of the patient and improve eyelid function.

Future studies are needed to understand the principles of using Dermatix SG for hypertrophic scars of complex anatomical structures to improve functional and aesthetic outcomes.

## Conclusions

This case demonstrates the effective use of Dermatix SG in improving both the aesthetic appearance and functionality of a traumatic hypertrophic scar on the eyelid. While SG is widely used for scar management, its application in traumatic scars affecting complex anatomical structures like the eyelids remains underexplored.

Our findings suggest that Dermatix SG not only improves scar appearance but also restores eyelid function, reducing discomfort and the risk of ocular complications. Further research is needed to optimise SG use in complex anatomical structures, potentially reducing the need for additional surgical interventions and enhancing both aesthetic and functional outcomes. We believe the product and its deployment deserve more attention and should be addressed by studies of greater relevance and magnitude.
